# Supplementary figures and images for: Co-infection of Candidatus Piscichlamydia Trichopodus (Order Chlamydiales) and Henneguya sp. (Myxosporea, Myxobolidae) in Snakeskin Gourami Trichopodus pectoralis (Regan 1910)
Source: Front Vet Sci. 2022 Mar 9;9:847977. doi: 10.3389/fvets.2022.847977 (PMC8961658; doi:10.3389/fvets.2022.847977)

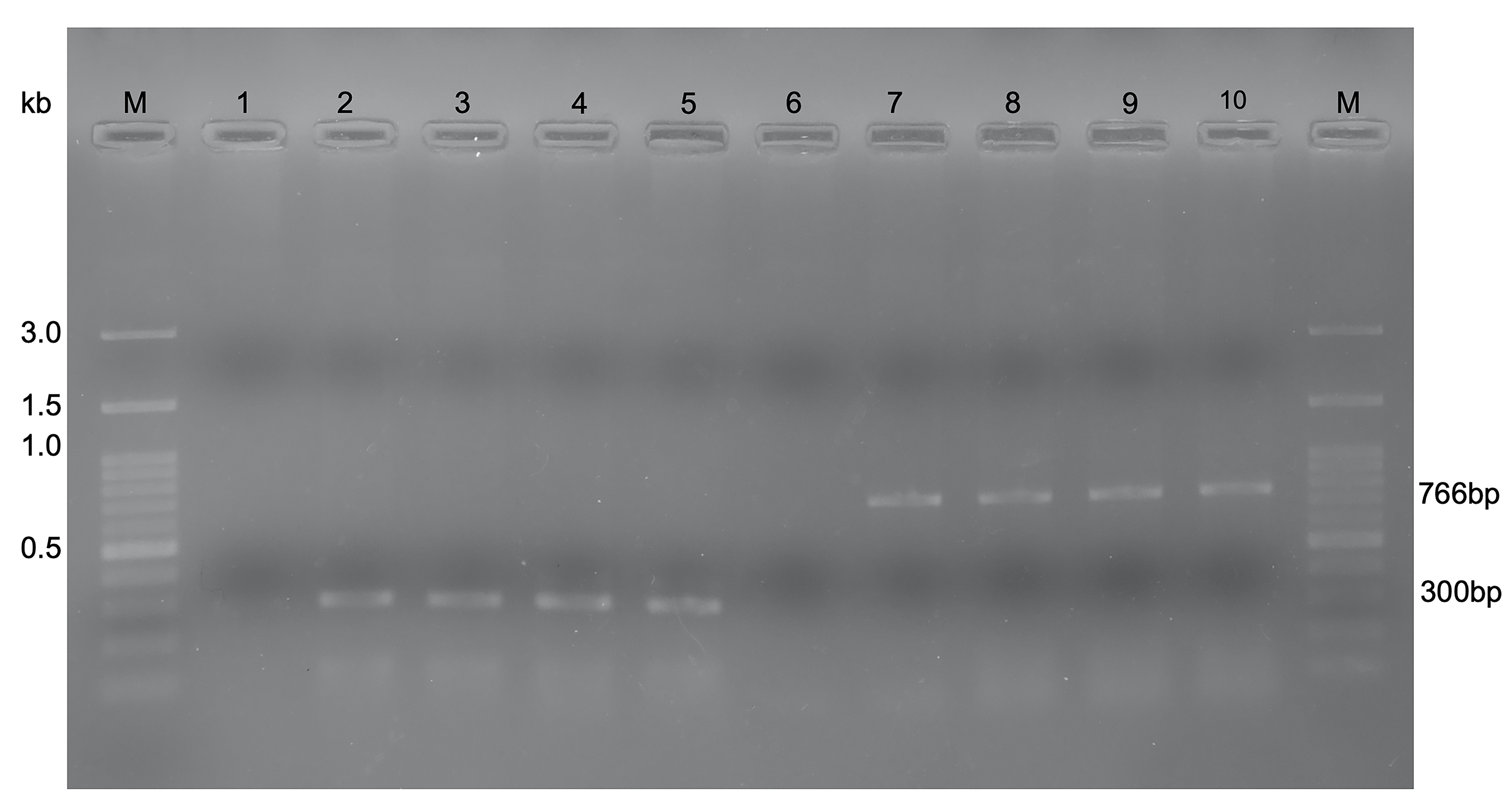

Supplement: Supplementary Figure S1 — Confirmation of PCR test results under agarose gel electrophoresis of samples from four representative fish. Lanes 1 and 6 were amplified without DNA template using primer set 1 and 2, respectively, as negative controls. Lanes 2, 3, 4, and 5 were amplified with the DNA template from primer set 1 (300 bp). Lanes 7, 8, 9, and 10 were amplified with the DNA template from primer set 2 (766 bp). Lane M was a DNA marker (Himedia, India). [file Image_1.JPEG]
